# Digital literacy and its effects on older adults’ health: exploring mechanisms and outcomes

**DOI:** 10.3389/fpubh.2025.1669425

**Published:** 2025-12-10

**Authors:** Shu Yang, Wenjie Xu, Kang Chen

**Affiliations:** 1 School of Physical Education, Northwest University for Nationalities, Lanzhou, China; 2 School of Sports Science and Physical Education, Southwestern University of Finance and Economics, Chengdu, China

**Keywords:** digital literacy, older adults, physical and mental health, social support, healthy lifestyles

## Abstract

**Introduction:**

In response to population aging, the Chinese government has prioritized enhancing the age-friendly adaptation of digital technologies, which have become essential for promoting social engagement and delivering health services to older adults. This study investigates whether digital literacy effectively improves physical and mental health in the aging population.

**Methods:**

Using longitudinal data from the China Family Panel Studies (CFPS, 2016–2022), we employ two-way fixed effects models and mechanism analysis to empirically examine the impact of digital literacy on health outcomes, while identifying underlying mechanisms and heterogeneous effects.

**Results:**

Key findings reveal that: (1) Digital literacy significantly improves both physical and psychological health among older adults. (2) These health gains are primarily mediated through enhanced social support networks and the adoption of healthier lifestyles. (3) The health benefits exhibit significant variation across educational attainment, gender, and health status.

**Discussion:**

The findings confirm that digital literacy is a significant determinant of healthy aging. They offer novel theoretical and empirical insights for policymakers, highlighting the importance of promoting digital literacy among older adults, particularly through interventions that leverage social support and healthy lifestyles.

## 1 Introduction

Declining fertility rates and increasing life expectancy have made population aging a major global challenge. According to the World Health Organization (WHO), the global demographic structure is increasingly shifting toward older age groups.^1^ Although population aging first emerged in high-income countries, low- and middle-income countries are now experiencing the most rapid demographic shifts. It is projected that by 2050, the global population aged 60 and over will reach 2.1 billion, with more than two-thirds of these older adults residing in low- and middle-income countries (see Footnote 1). This trend underscores the urgency of ensuring that older individuals maintain their independence and a high quality of life—a critical issue requiring focused attention, particularly in developing regions.

China, in particular, hosts the largest population of older adults in the world and is aging at one of the fastest rates globally. Examining how China promotes healthy aging could offer valuable insights for other developing countries. According to the China Statistical Yearbook (2024), the population aged 60 and above surpassed 300 million for the first time in 2024, reaching 310 million and accounting for 22% of the total population.^2^ The decline in cognitive, motor, and sensory functions associated with aging has led to increasingly serious physical and mental health problems among older adults. The China Aging Development Report (2024) indicates that over 80% of older adults in China suffer from at least one chronic illness, while 26.4% experience some degree of depressive conditions.^3^ Due to the country’s vast population base, rapid aging pace, and the extended duration of age-related illnesses, the older population poses substantial challenges to China’s healthcare system. In this context, it is essential to promote awareness among older adults that they are primarily responsible for their own health and to encourage them to regard “maintaining physical function and the ability to live independently” as a key health objective. This has become a major focus of China’s strategy for proactively addressing population aging.

In alignment with this strategy, the Healthy China 2030 initiative highlights digital information technology as a crucial instrument for advancing aging-related services. It provides essential support for strengthening the health system for older adults and promoting social engagement among them. However, as a group often marginalized in the digital landscape, older adults face a significant digital divide in accessing, using, and benefiting from digital information technologies. The digital divide refers to disparities in access to information technology (1), manifesting in four specific aspects: material access, motivational access, skills access, and usage access. With the continuous development of infrastructure construction, van Dijk (1) points out that while the material gap is gradually narrowing with continued infrastructure development, disparities in digital motivation, skills and usage remain persistent and are, in some cases, widening. Therefore, greater attention should be directed to the divide in digital motivation, skills and usage (2, 3).

In this context, enhancing the digital literacy of older adults has become a crucial step toward bridging the divides in motivation, skills, and usage among this population. Digital literacy refers to the ability of individuals to access, comprehend, organize, and critically assimilate digital information. This concept encompasses not only the ability to operate digital devices but also the proficiency in utilizing digital devices effectively (4–6). Specifically, digital access serves as the foundation for residents’ participation and integration into the digital society, enabling them to connect to the Internet anytime and anywhere through digital devices, thereby ensuring access to digital information technologies. In contrast, digital use pertains to the methods and extent of engagement with digital information technology. An improved level of digital use facilitates comprehensive integration into the digital society, allowing individuals to access diverse and specialized digital devices and maximize the benefits offered by digital information systems.

To enhance the digital literacy of older adults, the Chinese government has launched a series of policy initiatives. These include the Implementation Program on Effectively Addressing the Difficulties of Older Persons in the Use of Intelligent Technology, the Work Program on Promoting High-quality Development of Aging-adapted Digital Information Technology, and the Outline of Action for Enhancing Digital Literacy and Skills of the Entire Population. These policies have achieved significant progress. As of December 2024, there will be 157 million Internet users aged 60 and above in China, representing 52.5% of that age group. In terms of digital adaptation, 2,577 websites and applications commonly used by older individuals have completed age-friendly modifications, and 47.4% of Internet users aged 60 and above are able to use mobile phone applications in “senior mode.”^4^ These developments indicate that older adults have become a significant segment of new Internet users in China. As aging-friendly digital services continue to improve, the use of digital devices by older adults is no longer limited to basic communication. Increasingly, digital platforms serve as essential sources of information, tools for consumption and payment, reflecting a steady improvement in the digital literacy of the older population.

Existing research has predominantly focused on measuring digital literacy among the general population and exploring its determinants (7, 8). Gilster (4) first defined digital literacy as the capacity to access, understand, organize, and critically assimilate digital information. Building upon this foundational definition, numerous scholars and institutions have proposed their own interpretations. For instance, Sala et al. (9) argued that digital literacy should encompass both the content and the critical application of information and communication technology across work, leisure, learning, and communication contexts. Reddy et al. (6) defined digital literacy as the ability to search for, evaluate, and utilize information obtained through digital means, as well as to create and disseminate new content. Similarly, Chen et al. (10) emphasized that digital literacy includes the creative and critical use of digital technologies to access, assess, and share digital information. Although there is no universally accepted definition of digital literacy within the academic community, Ha and Kim (5), in their review of 735 relevant studies, found that terms such as “information,” “competence,” “assessment,” and “use” frequently appear in evaluations of digital literacy. This suggests that the core of digital literacy lies in whether individuals possess the ability to effectively use and critically assess digital information. However, few studies have specifically examined the health effects of digital literacy among older adults in China. Some studies have utilized China Family Panel Studies (CFPS) data to investigate the impact of digital literacy on individual physical health, with only brief mention of its effects on older adults within heterogeneity analyses (11, 12). Other studies have explored the influence of digital literacy on older adults’ healthcare-seeking habits or wellbeing, for example, Xia and Zhu (13) utilized data from the 2020 China Longitudinal Aging Social Survey (CLASS 2020) to investigate the impact of digital literacy on older adults’ utilization of community home care services, finding that enhanced digital literacy significantly reduced the probability of older adults using such services. Xin et al. (14), drawing on cross-sectional survey data from eastern, central, and western China, reported that digital literacy contributes to improved well-being among the older population. Broader studies also suggest that digital literacy improves healthcare service effectiveness (15), and is a significant determinant of population health (16), with benefits often contingent on well-developed digital infrastructure (17).

Still, some constraints in the current literature deserve note. First, most studies measure digital literacy primarily in terms of device access and operational skills, thus overlooking the motivational divide that older adults experience. According to digital divide theory (1–3), motivation is a critical dimension, especially for older adults, who may exhibit “digital distrust” (18). Concerns about information security, reliability, and accuracy can diminish their willingness to engage with digital services (19). Therefore, when assessing older adults’ digital literacy, consideration should not be limited to their access to and use of digital devices; attention must also be paid to their motivation regarding digital information technology. Second, although some studies have touched on healthcare behaviors or general well-being (13, 14), the direct impact of digital literacy on the physical and mental health of older adults remains inadequately studied. Existing research has examined the beneficial effects of digital literacy on individual health (11, 12), the direct impact of digital literacy on the physical and mental health of older adults requires further thorough investigation. Thirdly, it is essential to thoroughly investigate the mechanisms through which digital literacy enhances the physical and mental wellbeing of older adults. While existing research has explored the mechanisms by which digital literacy improves individual physical health, conclusions drawn from overall sample populations may not fully explain how digital literacy benefits the physical and mental wellbeing of older adults. For instance, Yang et al. (12) found that digital literacy primarily enhances individual health by increasing income, improving employment quality, and expanding social support. Given that most older adults have withdrawn from the labor market, it is necessary to delve deeper into the mechanisms by which digital literacy improves their physical and mental health.

To address these gaps, this study constructs a multidimensional digital literacy index based on data from the CFPS from 2016 to 2022. The index incorporates three dimensions—material access, motivation, and usage skills—to provide a more comprehensive assessment. Using this measure, we empirically examine the impact of digital literacy on the physical and mental health of older adults and investigate two underlying mechanisms: strengthened social support and the adoption of healthier lifestyles. Additionally, we explore heterogeneous effects across subgroups defined by educational attainment, gender, and baseline health status. Compared with the existing literature, this study makes the following marginal contributions. Firstly, it integrates the digital divide framework with the concept of digital literacy, constructing a digital literacy index from three dimensions: material access, motivation, and usage skills. Existing literature primarily measures digital literacy through access to digital devices and usage, without adequately considering older adults’ motivation for using digital devices (13, 14, 17). This study constructs a digital literacy index centered on three dimensions: material, motivation and usage skills. This aligns with the perspectives of van Dijk (1) and Deursen and Helsper (2), who contend that digital access should not solely focus on the acquisition of digital devices, but also on attitudes toward digital devices and usage skills. Second, this study employs a panel data to analyze the impact of digital literacy on the physical and mental health of older adults. Existing research has predominantly relied on cross-sectional data to examine digital literacy’s influence on healthcare choices or general well-being (13, 14), while other studies have explored its relationship with physical health in broader populations (11, 12). However, little attention has been given to its dual effect on both physical and mental health among older adults in China, particularly during the nation’s transition into a moderately aged society. Our longitudinal design helps fill this gap by providing more robust and dynamic evidence on this relationship. Finally, this paper contributes to the literature by systematically examining the mechanisms through which digital literacy may enhance physical and mental health, focusing on improved social support and the adoption of healthier lifestyles. This study aims to determine whether digital literacy can foster health improvements by enhancing interpersonal support networks and encouraging more health-conscious behaviors, thereby advancing the goal of healthy aging and offering new empirical evidence to inform policy and practice.

## 2 Literature review and research hypotheses

### 2.1 Digital literacy and the physical and mental health of older adults

The ongoing advancement of digital information technology has enabled modern tools to perform increasingly sophisticated and nuanced functions, allowing them to better address the diverse needs of individuals. In this evolving context, the digital divide is no longer confined to disparities in access to technology, but also encompasses differences in the ability to effectively use these tools to derive meaningful benefits. As van Dijk (1) observed, compared to the more visible gaps in device ownership or internet connectivity, the subtler divides related to motivation toward and usage of technology deserve greater attention. Consequently, the central issue in digital divide research has shifted toward understanding the disparities in outcomes resulting from differing attitudes and technological engagement (2). Within this framework, digital literacy has emerged as a critical factor shaping an individual’s capacity to capitalize on digital technologies. Existing literature on constructing and evaluating digital literacy typically centers its assessment on skills related to accessing and using digital devices (10, 17, 20–22), yet this approach often overlooks the critical dimension of motivation, particularly among older adults. Factors such as computer anxiety and technophobia can lead to “digital distrust” in this population, weakening their willingness to engage with digital technologies (1–3, 18), which in turn inhibits the development of their digital literacy. Empirical evidence confirms that motivation significantly influences older adults’ digital capabilities (7), with those showing stronger interest in digital platforms exhibiting greater confidence in their skills (23) and a stronger perception of the usefulness of online health information (24), thereby fostering more active engagement within the digital society. Based on this, we explore the role of digital literacy in enhancing the physical and mental health of older adults across three dimensions: material access, motivation, and usage skills.

In terms of material access, extensive research indicates that using digital devices can significantly improve older adults’ well-being (25–27). These improvements are often achieved through mechanisms such as strengthened social interactions and the promotion of healthier habits, with the effects being especially pronounced among older adults with fewer chronic conditions or those cohabiting with children (28, 29). Using digital devices has also been shown to foster improvements in physical and mental health by enhancing social trust and facilitating intergenerational communication (30, 31). Regarding motivation, maintaining an open and inclusive mindset toward digital platforms allows older adults to better leverage online information and services, which in turn supports their health. Buse (32) suggested that older adults’ hesitancy may stem from perceptions formed earlier in life, when devices like mobile phones were primarily associated with leisure, fostering distrust of virtual spaces (33). Concurrently, concerns about identity theft often lead to avoidance of online activities (34). In contrast, those with more positive attitudes are more likely to use digital devices effectively (35), hold more positive perceptions of aging (35), and maintain better physical and mental health (36). As for usage skills, proficiency in using digital devices helps older adults maintain independence, social connections, and health literacy, thereby supporting their overall quality of life (37, 38). Deursen and Helsper (2) found that among older adults, those aged 75 and above exhibited more limited internet usage patterns compared to their 65-74-year-old counterparts, with the majority employing it primarily for information retrieval. Consequently, diverse usage skills enable sustained engagement across multiple online platforms. On one hand, this facilitates the critical evaluation of health information, supporting better lifestyle decisions (17). On the other hand, broader social connections can strengthen social networks, buffer the impact of stress, and ultimately improve physical and mental well-being (39). Based on the above analysis, the following hypotheses are proposed:

*Hypothesis* 1: Digital literacy can improve the physical and mental health of older adults.

*Hypothesis* 1a: Enhancing older adults' material access can improve their physical and mental health.

*Hypothesis* 1b: Increasing older adults' motivation to use digital devices can improve their physical and mental health.

*Hypothesis* 1c: Improving older adults' usage skills can improve their physical and mental health.

### 2.2 Digital literacy, social support for older people’s physical and mental health

Digital literacy can effectively enhance older adults’ ability to interact with and adapt to their social environment, improve social participation in later life, and thereby promote their physical and mental health. As individuals age, the size of their social networks tends to shrink, and network integrity is progressively weakened (40). Retirement leads to the contraction of work-based social networks and reduces the number of active social roles; however, the frequent occurrence of negative life events, such as declining physical mobility, bereavement, and widowhood, further diminishes the networks formed through family and close relationships. These dynamics increase the risk of social isolation and loneliness among older adults (41). Studies have demonstrated that older adults who lack adequate social support are more likely to experience depressive symptoms, face diminished functional health, and encounter a higher risk of premature mortality (42–45). Enhancing digital literacy can help mitigate this trend by strengthening intergenerational relationships and increasing older adults’ social capital, thereby counteracting the decline in social network size and improving physical and mental well-being.

First, improved digital literacy can significantly increase the likelihood of participating in online social interactions and offline group activities. This engagement facilitates the expansion of new social networks, the development of potential weak ties, and the accumulation of social capital. Nguyen et al. (46) found that older adults with higher digital literacy levels were better able to build both online and offline social capital, for example, by asking questions on social media and viewing photos of family, friends, and others. Rich social capital has been shown to enhance physical and mental health outcomes (47, 48). Positive social engagement can mitigate the effects of daily stressors and stressful events, reduce the likelihood of adopting unhealthy behaviors, and increase physical activity frequency (114), thereby improving physical health (49). Moreover, socially active individuals tend to have greater trust in their community and society, leading to a stronger sense of social identity and significantly improved mental health (50).

Second, digital literacy enhances intergenerational ties between older adults and their children, increases intergenerational support, and raises the likelihood of monetary transfers from children to their parents (51). Stronger intergenerational connections are significantly associated with better physical and mental health outcomes (52). Older adults with robust intergenerational ties exhibit improved functional abilities, such as stronger grip strength and faster walking and stair-climbing speeds. These connections also increase the probability of engaging in physical activity and positively affect physical health (53). Furthermore, strong intergenerational bonds contribute to enhanced cognitive function, elevated subjective well-being, and improved mental health among older adults (52, 54). In summary, this paper proposes the following hypotheses:

*Hypothesis* 2: Digital literacy can improve the physical and mental health of older adults by enhancing their social support.

*Hypothesis* 2a: Digital literacy can improve the physical and mental health of older adults by increasing their social capital.

*Hypothesis* 2b: Digital literacy can improve the physical and mental health of older adults by increasing their intergenerational connections.

### 2.3 Digital literacy, healthy lifestyles, and the physical and mental health of older people

Digital literacy can effectively expand older adults’ access to health information and services, thereby encouraging the adoption of healthier lifestyles and improving physical and mental health. Xiong et al. (55) found that older adults search for health information online more frequently than younger individuals, and that the content they access is primarily oriented toward health science education and behavior improvement. Similarly, König et al. (28) observed that older adults disproportionately benefit from the health-promoting effects of digital literacy, as higher levels of digital literacy significantly improve their ability to locate and evaluate health-related information.

With high digital literacy, older adults are better able to utilize both the informational and instrumental functions of digital information technology to synthesize and compare multiple sources, enabling them to access more accurate and comprehensive health information (17). This information richness supports the development of healthier behaviors, as adequate health knowledge allows individuals to recognize potential health risks in daily life and avoid harmful behaviors (39, 41, 56). Specifically, older adults with broader access to health information are more likely to participate in healthcare practices (57), undergo physical examinations and screenings more frequently (58), maintain more diverse diets (59), and engage in higher levels of daily physical activity (60).

To enhance older adults’ awareness of essential health knowledge and promote healthier lifestyles, the Chinese government has developed extensive online platforms for health education. Through microblogs, Weibo, short videos, and other digital media, these platforms disseminate geriatric health information on nutrition, exercise, mental health, and the prevention of sensory, motor, and cognitive decline. Given older adults’ reliance on authoritative sources, such online health content effectively encourages informed health decisions and healthier behavior adoption (17, 61).

Empirical studies have consistently shown that healthy lifestyles, such as abstaining from smoking and alcohol and engaging in regular exercise, reduce the risk of all-cause mortality and extend life expectancy (62, 63). Conversely, unhealthy lifestyles represent the primary risk factor for premature death (64), contributing to at least 60% of early mortality and reducing life expectancy by 7.4 to 17.9 years (65). Transitions toward healthier behaviors are associated with an average increase in life expectancy of 1.19 years (66). In addition, adopting a healthy lifestyle is effective in reducing psychological distress and suicidal ideation, while also enhancing subjective well-being among older adults (67), thereby improving mental health outcomes. In summary, this paper proposes the following hypotheses:

*Hypothesis* 3: Digital literacy promotes the adoption of healthier lifestyles by older adults, thereby improving their physical and mental health.

*Hypothesis* 3a: Digital literacy reduces the likelihood that older adults will smoke and drink, thereby improving their physical and mental health.

*Hypothesis* 3b: Digital literacy increases the likelihood and frequency of regular exercise among older adults, thereby improving their physical and mental health.

**Figure 1:**
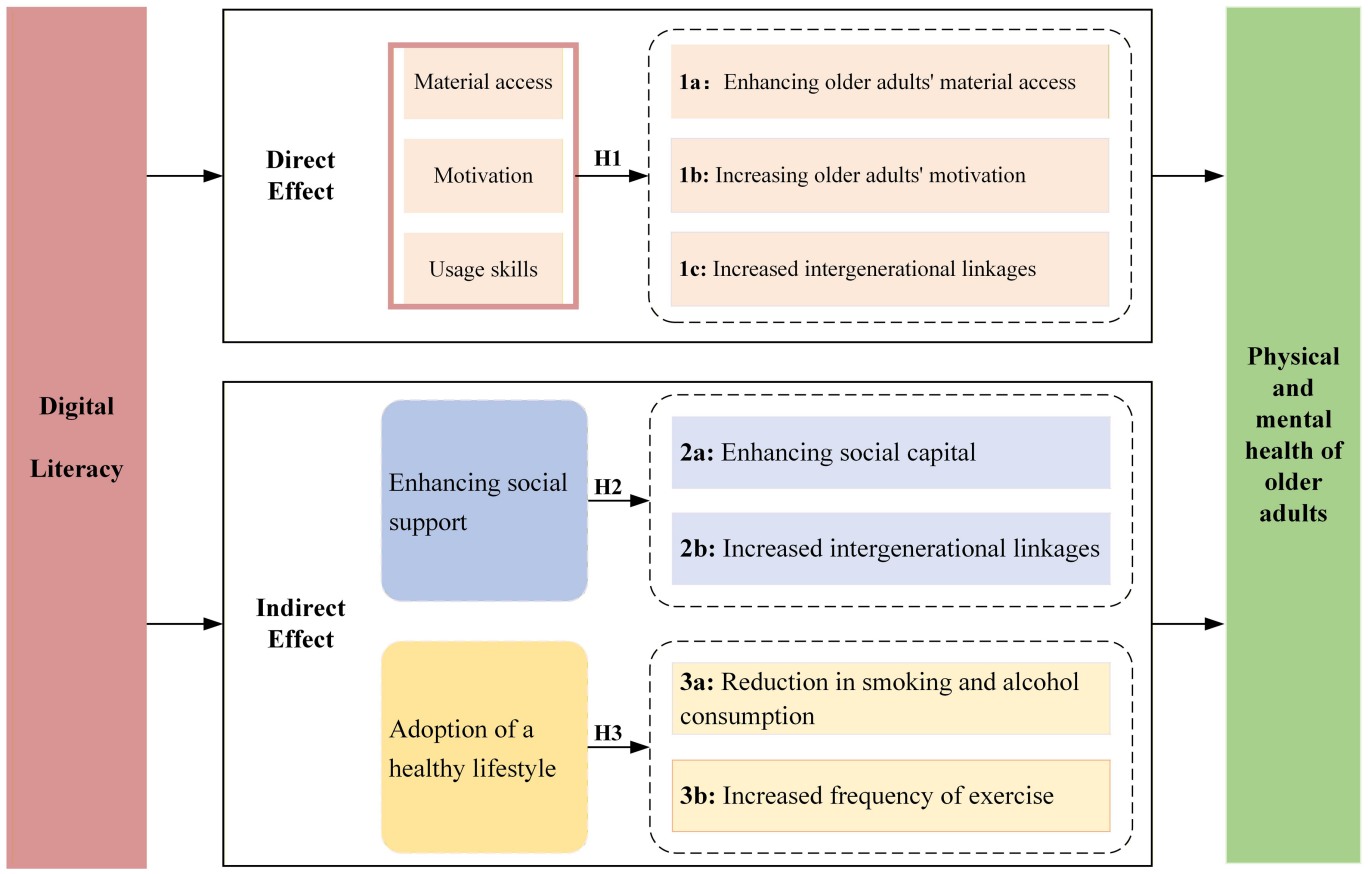
Mechanisms of digital literacy to improve the physical and mental health of older adults.

The mechanism of action of digital literacy in improving the physical and mental health of older adults is illustrated in Figure 1.

## 3 Research methods and data

### 3.1 Data sources

This paper utilizes data from the CFPS, developed by the Center for Social Science Surveys at Peking University. The CFPS conducted seven waves of surveys between 2010 and 2022, collecting longitudinal data on household consumption, income, and family relationships in China, thereby providing a comprehensive reflection of household economic behavior and population mobility. The CFPS employs a three-stage sampling method and covers 28 provinces, municipalities, and autonomous regions, making it a nationally representative dataset. As the CFPS has consistently adopted the Center for Epidemiologic Studies Depression (CES-D) scale developed by Radloff (68) to assess respondents’ mental health since 2016, this study selects the adult sample from 2016 to 2022 to ensure temporal comparability of mental health indicators. Based on this, the following sample screening procedures were applied: (1) exclusion of individuals aged below 60 or above 90; (2) removal of observations with extensive missing data on core variables; (3) weight the original sample using the longitudinal weights provided by the CFPS database’s individual panel to ensure that samples from different years possess national representativeness and inter-year comparability; and (4) trimming of the top and bottom 1% of continuous variables to mitigate the influence of outliers.

### 3.2 Variable settings

#### 3.2.1 Physical and mental health of older adults

In this study, the health of older adults is assessed along two dimensions: physical health and mental health. For physical health, the Activities of Daily Living Limitations (ADL) scale is used to evaluate an individual’s basic self-care capacity for independent living (51, 69–71). The CFPS assesses older adults’ self-care abilities across seven activities: independent outdoor activities, eating, kitchen tasks, using public transport, shopping, personal hygiene, and laundry. One point is given for each activity performed independently, yielding a total ADL score ranging from 1 to 7, with higher scores indicating stronger self-care ability and better physical health. For mental health, the study uses respondents’ scores on the CES-D scale (72–74). Since 2016, the CFPS has adopted an abbreviated 8-item version of the CES-D scale. The items cover six negative emotions—feeling down, worthless, troubled by sleep, lonely, sad, or unable to cope with life—and two positive emotions: feeling cheerful and happy with life. In scoring, responses to negative emotion items are recorded on a Likert scale, while the positive items are reverse-scored. These are summed to generate a total CES-D score, with higher values indicating a greater tendency toward depression. This study uses the CES-D scores as provided directly in the CFPS questionnaire to maintain data consistency.

#### 3.2.2 Digital literacy

Drawing on the framework of the digital divide and the concepts of digital literacy (1–3, 10, 17, 18, 20–22), we constructed a comprehensive digital literacy (DL) index using data from the CFPS. The index incorporates three dimensions: material access, motivation, and usage skills (Table 1). Material access focuses on whether older adults can utilize digital information technology devices to access the internet; motivation centers on their subjective initiative in employing digital information technology; usage skills emphasize their capacity to utilize digital information technology. Considering data availability, 10 indicators were ultimately selected to measure digital literacy among China’s older adults. Internal consistency reliability was assessed using Cronbach’s alpha, yielding a value of 0.887, indicating excellent reliability.

**Table 1:** The construction of digital literacy.

Dimensions	Primary indicators	Secondary indicators	Definition	Calculation	DL
Material access	DL_material	DL_material	Use mobile phones or computers to access the Internet, yes = 1, no = 0	Standardized	The standardized values of DL_material, DL_motivation, and DL_us were summed to yield DL.
Motivation	DL_motivation	DL_motivation	The importance of the internet for work, study, entertainment, social interaction, and commercial activities.	Averaged and standardized	
Usage skills	DL_us	us_study	Engage in internet learning, yes = 1, no = 0	Summarized and standardized	
		us_joy	Engage in internet entertainment, yes = 1, no = 0		
		us_social	Network socially, yes = 1, no = 0		
		us_buy	Engage in online shopping, yes = 1, no = 0		

For material access (DL_material), we measured whether respondents used a computer or smartphone to access the internet, assigning a value of 1 if yes and 0 otherwise. For motivation (DL_motivation), it was assessed using five CFPS items that asked respondents to rate the importance of the internet across different domains—work, study, entertainment, social interaction, and commerce—on a 5-point Likert scale; responses were averaged to form a composite motivation score. For usage skills (DL_us), the CFPS asked respondents whether they used the internet for learning (us_study), entertainment (us_joy), social interaction (us_social), and commercial activities (us_buy). Each activity was coded as 1 if performed and 0 otherwise. We summed these four binary variables to create a usage diversity score. To ensure equal weighting across the three equally critical dimensions of digital literacy, we standardized the scores for material access, motivation, and usage skills (mean = 0, SD = 1) and summed them to create the overall digital literacy index.

We also conducted robustness checks using a factor-analytic approach to construct the digital literacy index. The high KMO value (0.842) and significant Bartlett’s test (*p* < 0.01) confirmed the factorability of our data. Nevertheless, we maintained the composite index (sum of standardized dimensions) as the primary measure of digital literacy, because it aligns with our conceptual framework and facilitates straightforward interpretation of how each digital literacy component affects health outcomes.

#### 3.2.3 Mechanism variables

The level of social support received by older adults is measured through two components: social capital and intergenerational ties. For social capital, offline and online social capital are captured using two variables: household expenditure on favors and frequency of Internet-based socializing, respectively. Expenditure on favors and gifts, which is defined as spending on gift exchanges during festivals, weddings, and funerals, effectively reflects the size and strength of kinship-based social networks among older adults (75, 76). To reduce the influence of outliers and mitigate potential multicollinearity, this variable is log-transformed. The frequency of using the Internet for social interaction reflects the extent of older adults’ online engagement and serves as an indicator of accumulated online social capital (27). For intergenerational ties, this study uses two variables: the frequency of meeting with children and the frequency of contact with children. Together, these metrics capture the strength of intergenerational relationships between older adults and their offspring (54).

To assess whether older adults maintain healthy habits related to smoking, alcohol consumption, and physical activity, the CFPS records whether respondents smoke cigarettes, drink alcohol more than three times per week, and whether they have engaged in physical activity in the past month. These indicators are used to evaluate whether digital literacy reduces the likelihood of smoking and drinking, and increases both the likelihood and frequency of physical activity among older adults.

#### 3.2.4 Control variables

We selected control variables spanning three dimensions: individual characteristics, socioeconomic attributes, and living environments. At the individual level, we controlled for age, gender, and marital status. Existing studies show that gender influences older adult health in complex ways. While women tend to live longer, men often exhibit better self-rated health, mental well-being, and lower chronic disease prevalence (49, 77, 78). Similarly, marital status significantly shapes health outcomes. Widowhood not only impairs mental health but also elevates risks of chronic illness and functional decline by disrupting healthy lifestyles (79). Moreover, living alone has been linked to better physical health yet higher depression levels (71). Regarding socioeconomic attributes, we included education, retirement status, pension receipt, wage level, and household per capita income. Consistent with prior work, higher education and income are generally associated with better physical and mental health in older adults (70, 80, 81). Finally, in terms of living environments, we controlled for urban–rural residence, which captures regional disparities in economic development, infrastructure, and healthcare access—all critical determinants of older adult well-being (7, 82). Definitions of variables and descriptive statistics are presented in Table 2.

**Table 2:** Variable definitions and descriptive statistics.

Variables	Variable definition	N	Mean	S.d.
Core variables				
ADL	Degree of limitation of the ability to perform activities of daily living (ADLs), with values ranging from 1 to 7; the greater the value obtained, the less limited life is.	30,766	6.271	1.547
MH	Mental health status score, with higher scores indicating a higher tendency to depression.	25,453	33.64	9.110
DL	Digital literacy, summed from the standardized DL_material, DL_motivation and DL_us.	32,524	0.000	2.723
DL_material	Use mobile phones or computers to access the Internet, yes = 1, no = 0	32,524	0.130	0.336
DL_motivation	Motivation for using digital information technology	32,524	0.425	1.130
DL_us	Skills in using digital infromation technology	32,524	0.518	1.038
us_study	Engage in internet learning, yes = 1, no = 0	32,524	0.024	0.152
us_joy	Engage in internet entertainment, yes = 1, no = 0	32,524	0.095	0.293
us_social	Network socially, yes = 1, no = 0	32,524	0.131	0.337
us_buy	Engage in online shopping, yes = 1, no = 0	32,524	0.034	0.181
Mechanism variables				
Offline social capital	Expenditure on favors (Yuan/year), in logarithms	31,375	6.663	2.968
Online social capital	Frequency of social internet use, never = 1, once every few months = 2, once a month = 3, 2–3 times a month = 4, 1–2 times a week = 5, 3–4 times a week = 6, almost every day = 7	4,704	3.250	2.429
Frequency of meeting	Frequency of meeting with children, never = 1, once every few months = 2, once a month = 3, 2–3 times a month = 4, 1–2 times a week = 5, 3–4 times a week = 6, almost every day = 7	32,524	3.151	2.414
frequency of contact	Frequency of contact with children, never = 1, once every few months = 2, once a month = 3, 2–3 times a month = 4, 1–2 times a week = 5, 3–4 times a week = 6, almost every day = 7	32,524	3.130	2.427
nonsmoking	Smoked in the past month, yes = 0, no = 1	25,597	0.726	0.446
non-alcoholic	Drank more than 3 times per week in the past month, yes = 0, no = 1	25,596	0.837	0.369
Exercise	Exercise habit, yes = 1, no = 0	32,524	0.755	0.430
Exercise frequency	Average number of workouts per week	29,872	4.445	3.420
Control variables				
Age	Age	32,524	69.58	7.109
Gender	female = 0, male = 1	32,522	0.485	0.500
Edu	Educational level, illiterate = 1, primary school = 2, lower secondary school = 3, upper secondary school = 4, college = 5, undergraduate degree = 6, master’s degree and above = 7	29,706	1.971	1.143
Marriage	Married = 1, unmarried (including widowed and divorced) = 0	30,234	0.813	0.390
Retirement	Retired = 1, not retired = 0	32,458	0.595	0.491
Pension	Pension collection processed = 1, not processed = 0	32,524	0.609	0.488
Income	Income in the last 12 months (10,000 yuan)	32,524	0.149	0.701
Per capita	Per capita net family income (10,000 yuan)	31,761	2.123	2.406
Urban	Rural = 0, urban = 1	31,928	0.470	0.499

DL_material, DL_motivation, and DL_us are the original values. After standardization, they are summed to yield DL. For multidimensional regression, standardized data from each dimension is employed to ensure horizontal comparability across dimensions.

### 3.3 Model

#### 3.3.1 Benchmark regression model

To examine the health effects of digital literacy, we employ a two-way fixed effects model, in line with existing literature (22, 51, 83). This model accounts for unobserved, time-invariant differences across individuals and captures aggregate trends over time that affect all individuals, thus reducing concerns about omitted variables. The empirical model takes the following form:


$$\text{Healt}h_{\mathrm{it}}=\alpha_{0}+\alpha_{1}DL_{\mathrm{it}}+\alpha_{2}X_{\mathrm{it}}+\mu_{i}+\theta_{t}+\varepsilon_{\mathrm{it}}$$ (1)

Here, $\text{Healt}h_{\mathrm{it}}$ denotes the health status (both physical and mental) of older adult *i* in year *t*. $DL_{\mathrm{it}}$ represents the digital literacy level of the same individual in the corresponding year. $X_{\mathrm{it}}$ is a vector of control variables. To mitigate bias arising from unobserved individual heterogeneity or time-specific effects, individual fixed effects $\mu_{i}$ and time fixed effects $\theta_{t}$ are included. $\varepsilon_{\mathrm{it}}$ is the random error term. If the coefficient $\alpha_{1}$ is significantly positive, it indicates that digital literacy has a beneficial effect on the physical and mental health of older adults.

#### 3.3.2 Instrumental variables method

To address the potential issue of reverse causality between digital literacy and the health status of older adults, namely, that healthier individuals may be more inclined to engage with new technologies and thus exhibit higher digital literacy, this study employs an Instrumental Variable (IV) method to further examine the causal effect of digital literacy on health outcomes:


$$DL_{\mathrm{it}}=\lambda_{0}+\lambda_{1}IV_{\mathrm{it}}+\lambda_{2}X_{\mathrm{it}}+\mu_{i}+\theta_{t}+\varepsilon_{\mathrm{it}}$$ (2)

$$\begin{array}{c} \text{Healt}h_{\mathrm{it}}=\rho_{0}+\rho_{1}{\hat{\mathrm{DL}}}_{\mathrm{it}}+\rho_{2}X_{\mathrm{it}}+\mu_{i}+\theta_{t}+\varepsilon_{\mathrm{it}} \end{array}$$ (3)

In Equation 2, $IV_{\mathrm{it}}$ serves as the instrumental variable for digital literacy. First, the estimated value of digital literacy ${\hat{\mathrm{DL}}}_{\mathrm{it}}$ is obtained via Equation 2, then Equation 3 is employed to estimate the impact of digital literacy on the physical and mental health of older adults. The meanings of the remaining variables are identical to those in Equation 1.

#### 3.3.3 Mechanism model

As discussed earlier, digital literacy may influence the physical and mental health of older adults by enhancing social support and promoting the adoption of healthier lifestyles. To test this mediation pathway, a mediated effects model is employed to assess whether improvements in health outcomes among older adults are attributable to increased social support and healthier behavior patterns (84–86).


$$\begin{array}{c} \text{Socia}l_{\mathrm{it}}=\beta_{0}+\beta_{1}DL_{\mathrm{it}}+\beta_{2}X_{\mathrm{it}}+\mu_{i}+\theta_{t}+\varepsilon_{it} \end{array}$$ (4)

$$\begin{array}{c} \text{Prob}(\text{lifestyl}e_{\mathrm{it}}=1)=\Phi (\delta_{0}+\delta_{1}DL_{\mathrm{it}}+\delta_{2}X_{\mathrm{it}}+\mu_{i}+\theta_{t}+\varepsilon_{\mathrm{it}}) \end{array}$$ (5)

$$\begin{array}{c} \begin{array}{l} \text{Healt}h_{\mathrm{it}}=\gamma_{0}+\gamma_{1}DL_{\mathrm{it}}+\gamma_{2}\text{socia}l_{\mathrm{it}}/\text{lifestyl}e_{\mathrm{it}}+\\ \gamma_{3}X_{\mathrm{it}}+\mu_{i}+\theta_{t}+\varepsilon_{\mathrm{it}} \end{array} \end{array}$$ (6)

In Equation 4, $\text{Socia}l_{\mathrm{it}}$ represents the level of social support received by older adult *i* in year *t*. Given that healthy lifestyle variables are binary, a Probit model is adopted in Equation 5 to evaluate the impact of digital literacy on lifestyle behaviors, where $\text{lifestyl}e_{\mathrm{it}}$ denotes the health-related lifestyle of older adult *i* in year *t*. The definitions of the remaining variables are consistent with those in Equation 1. By jointly analyzing Equations 4, 6, we test whether digital literacy enhances health outcomes through increased social support. Specifically, if both $\beta_{1}$ and $\;\gamma_{2}$ are statistically significant, it is concluded that digital literacy improves health by enhancing social support. Similarly, Equations 5, 6 are used to verify whether digital literacy contributes to better health outcomes by promoting a healthier lifestyle. If both $\delta_{1}$ and $\gamma_{2}$ are significant, the mediation effect through lifestyle is considered valid.

## 4 Empirical analysis

### 4.1 Impact of digital literacy on the health of older adults

Table 3 presents the regression results from Model (1), where columns (1)–(3) report the effects of digital literacy on physical health, and columns (4)–(6) report its effects on mental health. Columns (3) and (6) incorporate both individual and time fixed effects in addition to the full set of control variables. Even after accounting for these factors, digital literacy continues to exhibit a significant positive effect on both the physical and mental health of older adults.

**Table 3:** Impact of digital literacy on physical and mental health of older adults.

Variables	(1)	(2)	(3)	(4)	(5)	(6)
	ADL	ADL	ADL	MH	MH	MH
DL	0.091***	0.031***	0.050***	−0.369***	−0.120***	−0.134***
	(0.003)	(0.003)	(0.005)	(0.022)	(0.027)	(0.028)
Age		−0.063***	−0.029		−0.017	−0.014
		(0.003)	(0.023)		(0.013)	(0.014)
Gender		0.113***	0.021		−2.276***	−2.368***
		(0.026)	(0.141)		(0.158)	(0.155)
Edu		0.051***	0.029		−0.556***	−0.516***
		(0.013)	(0.083)		(0.076)	(0.079)
Marriage		0.128***	0.018		−2.348***	−2.252***
		(0.038)	(0.063)		(0.232)	(0.231)
Retirement		0.162***	0.151***		−0.621***	−0.516***
		(0.028)	(0.044)		(0.167)	(0.171)
Pension		0.048*	0.068**		0.159	0.136
		(0.025)	(0.034)		(0.151)	(0.153)
Income		0.036***	0.017*		−0.147**	−0.126*
		(0.007)	(0.010)		(0.072)	(0.074)
Per capita		0.014***	0.003		−0.258***	−0.188***
		(0.004)	(0.004)		(0.030)	(0.031)
Urban		0.100***	−0.012		−1.387***	−1.106***
		(0.027)	(0.062)		(0.166)	(0.193)
Constant	6.264***	10.196***	8.089***	33.759***	40.643***	39.940***
	(0.013)	(0.177)	(1.635)	(0.077)	(0.976)	(0.990)
N	30,766	26,908	24,174	25,453	22,699	22,583
*R* ^2^	0.026	0.109	0.752	0.015	0.102	0.142
Ind. fixed effect	No	No	Yes	No	No	Yes
Time fixed effect	No	No	Yes	No	No	Yes

(1)***, **, and *denote 1, 5 and 10% significance levels, respectively. (2) Cluster robust standard errors for clustering at the individual level are in parentheses.

As shown in column (3), digital literacy significantly improves physical health. Specifically, each one-unit increase in digital literacy corresponds to a 0.05-point rise in physical health score, indicating an enhanced capacity for self-care in daily activities. Column (6) reveals a similar trend for mental health: each one-unit increase in digital literacy reduces the CES-D depression score by an average of 0.134 points, suggesting a meaningful improvement in mental health. This indicates that enhancing digital literacy can effectively improve the physical and mental health of older adults, thereby validating Hypothesis 1.

To examine the specific contributions of each digital literacy dimension, we disaggregated the composite measure and conducted separate regression analyses for material access, motivation, and usage skills. As reported in Table 4, all three dimensions show significant benefits for older adults’ health. Specifically, older adults with digital device access demonstrated significantly better physical health (Column 1) and mental health (Column 2) compared to non-users, supporting Hypothesis 1a. Similarly, stronger motivation to use digital information technology was associated with improved outcomes: each one-unit increase in motivation corresponded to a 0.124-point rise in physical health scores (Column 3) and a 0.273-point reduction in CES-D depression scores (Column 4), confirming Hypothesis 1b. Likewise, enhanced usage skills also contributed positively: a one-unit increase in the skills index led to a 0.116-point improvement in physical health and a 0.363-point decrease in depression scores. Building upon this, we further investigated whether all usage skills could enhance the physical and mental health of older adults (Table 5). We found that using digital devices for learning (Column 1), entertainment (Column 3), socializing (Column 5), and commercial activities (Column 8) effectively improved older adults’ physical health. However, using digital devices for learning (Column 2) and commercial activities (Column 8) failed to effectively improve their mental wellbeing. Thus, our findings do not conclusively support Hypothesis 1c. Not all usage skills can improve the physical and mental health of older adults.

**Table 4:** Impact of different dimensions of digital literacy on physical and mental health of older adults.

Variables	(1)	(2)	(3)	(4)	(5)	(6)
	ADL	MH	ADL	MH	ADL	MH
DL_material	0.080***	−0.124*				
	(0.010)	(0.071)				
DL_motivation			0.124***	−0.273***		
			(0.010)	(0.066)		
DL_us					0.116***	−0.363***
					(0.013)	(0.086)
Cons.	10.541***	−6.917	10.462***	39.734***	10.436***	39.026***
	(0.194)	(21.044)	(0.194)	(0.986)	(0.195)	(1.087)
N	26,470	19,196	26,470	22,583	26,470	22,252
*R* ^2^	0.202	0.681	0.204	0.141	0.203	0.216
Controls	Yes	Yes	Yes	Yes	Yes	Yes
Ind. fixed effect	Yes	Yes	Yes	Yes	Yes	Yes
Time fixed effect	Yes	Yes	Yes	Yes	Yes	Yes

(1)***, **, and *denote 1, 5 and 10% significance levels, respectively. (2) Cluster robust standard errors for clustering at the individual level are in parentheses. (3) DL_material, DL_motivation, and DL_us are standardized values.

**Table 5:** Impact of different usage skills on physical and mental health of older adults.

Variables	(1)	(2)	(3)	(4)	(5)	(6)	(7)	(8)
	ADL	MH	ADL	MH	ADL	MH	ADL	MH
us_study	0.152***	−0.426						
	(0.044)	(0.318)						
us_joy			0.210***	−0.928***				
			(0.032)	(0.208)				
us_social					0.310***	−0.789***		
					(0.030)	(0.199)		
us_business							0.188***	−0.447
							(0.041)	(0.301)
Cons.	10.619***	38.420***	10.469***	39.134***	10.294***	39.260***	10.583***	38.506***
	(0.193)	(1.072)	(0.195)	(1.090)	(0.198)	(1.101)	(0.194)	(1.078)
N	26,470	22,252	26,470	22,252	26,470	22,252	26,470	22,252
*R* ^2^	0.200	0.215	0.202	0.216	0.205	0.216	0.201	0.215
Controls	Yes	Yes	Yes	Yes	Yes	Yes	Yes	Yes
Ind. fixed effect	Yes	Yes	Yes	Yes	Yes	Yes	Yes	Yes
Time fixed effect	Yes	Yes	Yes	Yes	Yes	Yes	Yes	Yes

(1)***, **, and *denote 1, 5 and 10% significance levels, respectively. (2) Cluster robust standard errors for clustering at the individual level are in parentheses.

### 4.2 Robustness check

This paper conducts three types of robustness tests. First, it replaces the original measures of digital literacy, physical and mental health with alternative indicators. Second, to address potential reverse causality, an IV method is employed to re-estimate the impact of digital literacy on health outcomes. Third, additional fixed effects are included to reduce the risk of omitted variable bias.

#### 4.2.1 Variable substitution

To test the robustness of our findings, we conducted a series of alternative measurements. First, digital literacy was recalculated using factor analysis (DL_factor) and regressed on health outcomes. As shown in Columns (1) and (2) of Table 6, higher digital literacy is associated with a 0.481-point increase in physical health scores and a 0.916-point decrease in CES-D depression scores. Second, we substituted the health outcome variables: physical health was measured using a 5-point self-assessed health scale, while mental health was defined based on whether CES-D scores exceeded the 95th percentile, following Radloff (68). A Probit model was used to estimate the likelihood of depression. Results in Columns (3) and (4) confirm that digital literacy significantly improves self-rated health and reduces the probability of depression, with each unit increase in digital literacy corresponding to a 0.021-point rise in self-assessed health and a 0.2% decrease in depression risk. Together, these alternative approaches consistently support the positive effect of digital literacy on older adults’ health, reinforcing the reliability of our baseline estimates.

**Table 6:** Variable substitution.

Variables	(1)	(2)	(3)	(4)
	ADL	MH	Self-assessment of health	Presence of depressive symptoms
DL_factor	0.481***	−0.916***		
	(0.047)	(0.292)		
DL			0.021***	−0.002***
			(0.006)	(0.001)
Constant	9.000***	38.717***	6.476***	0.036
	(2.382)	(1.083)	(1.766)	(0.259)
N	19,607	22,252	24,251	27,787
*R* ^2^	0.672	0.215	0.609	
Controls	Yes	Yes	Yes	Yes
Ind. fixed effect	Yes	Yes	Yes	Yes
Time fixed effect	Yes	Yes	Yes	Yes

(1)***, **, and *denote 1, 5 and 10% significance levels, respectively. (2) Cluster robust standard errors for clustering at the individual level are in parentheses. (3) For ease of understanding, marginal effects are reported in column (4).

#### 4.2.2 IV method

Drawing from the CFPS database, the Internet penetration rate at the neighborhood committee (or village) level, which is calculated as the number of Internet users divided by the total population in the committee, is used as an IV for individual-level digital literacy (87, 88). Regarding instrument validity, a higher Internet penetration rate reflects more advanced local digital infrastructure and a stronger digital culture, satisfying the correlation condition. In terms of exogeneity, the Internet penetration rate is a contextual, macro-level variable that does not directly affect the individual health status of older adults, thereby meeting the exogeneity requirement. Table 7 presents the regression results using the IV method. Panel B reports the first-stage regression results, showing that the Internet penetration rate at the village level is significantly and positively associated with the digital literacy level of older adults at the 1% significance level. The corresponding F-statistics in all of the specifications exceed the conventional threshold of 10, indicating that the instrument is strong and that weak instrument bias is not a concern. As shown in columns (1) and (2), digital literacy continues to significantly improve ADL scores and reduce CES-D scores among older adults. These findings confirm that digital literacy has a positive effect on both physical and mental health, thereby reinforcing the robustness and credibility of the baseline regression results presented in this paper.

**Table 7:** Instrumental variables approach.

Variables	(1)	(2)
	ADL	MH
Panel A		
DL	0.091***	−0.430***
	(0.029)	(0.165)
Panel B	DL	DL
Internet penetration	4.515***	4.966***
	(0.224)	(0.235)
F-statistics	404.8	446.0
N	22,783	22,252
Controls	Yes	Yes
Ind. fixed effect	Yes	Yes
Time fixed effect	Yes	Yes

(1)***, **, and *denote 1, 5 and 10% significance levels, respectively. (2) Cluster robust standard errors for clustering at the individual level are in parentheses.

#### 4.2.3 Addition of fixed effects

Given the regional disparities in the development of digital information infrastructure across China, this study further controls for province-specific time trends to address potential omitted variable bias. As reported in Table 8, after accounting for these province-level time trends, the effect of digital literacy on the health of older adults remains significant. Specifically, for each one-unit increase in digital literacy, the physical health score increases by an average of 0.063 points (Column 1), indicating that older adults’ ability to live independently continues to improve. Additionally, the average score for depressive symptoms decreases by 0.105 points, reflecting a significant improvement in mental health. These results confirm that the core findings from the benchmark regression are robust and reliable, even after incorporating more detailed fixed effects.

**Table 8:** Addition of fixed effects.

Variables	(1)	(2)
	ADL	MH
DL	0.063***	−0.105***
	(0.006)	(0.029)
Constant	9.758***	51.657***
	(2.396)	(15.760)
Province × time	Yes	Yes
N	19,607	22,252
*R* ^2^	0.673	0.216
Controls	Yes	Yes
Ind. fixed effect	Yes	Yes
Time fixed effect	Yes	Yes

(1)***, **, and *denote 1, 5 and 10% significance levels, respectively. (2) Cluster robust standard errors for clustering at the individual level are in parentheses.

### 4.3 Mechanism testing

#### 4.3.1 Digital literacy, social support, and the physical and mental health of older adults

To assess whether digital literacy enhances the physical and mental health of older adults by increasing their social capital, a mediation effect model is employed (Table 9). As shown in column (1), digital literacy significantly increases household expenditure on favors and gifts. Specifically, for every one-unit increase in digital literacy, expenditure on favors and gifts rises by 5.2%, suggesting that enhanced digital literacy helps reinforce the proximate social networks of older adults. Combining the results from columns (1)–(3), it is evident that digital literacy improves physical health (Column 2) and mental health (Column 3) through the strengthening of proximate, kinship-based social capital. In Column (4), digital literacy is also shown to significantly increase the frequency of Internet-based social interaction. For each one-unit increase in digital literacy, online socializing frequency increases by an average of 0.318 points, indicating that digital literacy can effectively expand online social capital among older adults. Together, columns (4)–(5) demonstrate that digital literacy improves both physical and mental health by enhancing online social capital. These effects contribute to improvements in physical and mental health, thereby supporting Hypothesis 2a.

**Table 9:** Digital literacy, social capital, and older adults’ physical and mental health.

Variables	(1)	(2)	(3)	(4)	(5)	(6)
	Offline social capital	ADL	MH	Online social capital	ADL	MH
DL	0.052***	0.062***	−0.114***	0.318***	0.031*	−0.205***
	(0.009)	(0.006)	(0.027)	(0.017)	(0.016)	(0.068)
Offline social capital		0.010**	−0.157***			
		(0.004)	(0.023)			
Online social capital					0.020***	−0.096*
					(0.008)	(0.058)
Constant	8.156***	9.186***	42.196***	−0.341	2.792	42.686***
	(0.291)	(2.391)	(1.003)	(0.590)	(12.193)	(2.132)
N	27,021	19,337	22,465	4,375	2,243	4,415
*R* ^2^	0.237	0.676	0.104	0.293	0.669	0.125
Controls	Yes	Yes	Yes	Yes	Yes	Yes
Ind. fixed effect	Yes	Yes	Yes	Yes	Yes	Yes
Time fixed effect	Yes	Yes	Yes	Yes	Yes	Yes

(1)***, **, and *denote 1, 5 and 10% significance levels, respectively. (2) Cluster robust standard errors for clustering at the individual level are in parentheses.

To further examine whether digital literacy improves the physical and mental health of older adults by enhancing intergenerational contact (Table 10), the results from Column (1) and Column (4) indicate that digital literacy significantly increases the frequency of meetings and communication between older adults and their children. Specifically, each one-unit increase in digital literacy leads to an average increase of 0.176 in the frequency of meetings (Column 1) and 0.188 in the frequency of contact (Column 4). The synthesis of Table 10 confirms that digital literacy effectively strengthens intergenerational connections, which in turn improves the physical and mental health of older adults, thereby validating Hypothesis 2b. Tables 9, 10 collectively demonstrate that digital literacy can enhance social support and reduce the risk of social isolation among older adults, leading to significant improvements in their physical and mental health. These findings provide further validation for Hypothesis 2.

**Table 10:** Digital literacy, intergenerational connections, and physical and mental health in older adults.

Variables	(1)	(2)	(3)	(4)	(5)	(6)
	Frequency of meeting	ADL	MH	Frequency of meeting	ADL	MH
DL	0.176***	0.053***	−0.107***	0.188***	0.054***	−0.083***
	(0.009)	(0.005)	(0.028)	(0.010)	(0.006)	(0.029)
Frequency of meeting		0.064***	−0.342***			
		(0.008)	(0.041)			
Frequency of contact					0.057***	−0.285***
					(0.007)	(0.037)
Constant	6.684**	8.628***	40.080***	2.967	9.000***	40.576***
	(3.185)	(2.366)	(1.095)	(3.205)	(2.352)	(1.116)
N	24,636	19,607	22,252	24,636	19,607	22,252
*R* ^2^	0.702	0.675	0.219	0.702	0.675	0.218
Controls	Yes	Yes	Yes	Yes	Yes	Yes
Ind. fixed effect	Yes	Yes	Yes	Yes	Yes	Yes
Time fixed effect	Yes	Yes	Yes	Yes	Yes	Yes

(1)***, **, and *denote 1, 5 and 10% significance levels, respectively. (2) Cluster robust standard errors for clustering at the individual level are in parentheses.

#### 4.3.2 Digital literacy, healthy lifestyles, and the physical and mental health of older adults

To examine whether digital literacy improves the physical and mental health of older adults by promoting healthier lifestyles, Models (5) and (6) were tested (Table 11). As shown in column (1), digital literacy significantly reduces the likelihood of smoking among older adults. Specifically, for every one-unit increase in digital literacy, the probability of smoking in the past month decreases by 0.3%. Combining the results from columns (1)–(3) indicates that digital literacy can effectively improve physical and mental health by reducing the probability of smoking among the older adults. Column (4) demonstrates that digital literacy significantly reduces the frequency of alcohol consumption. For each one-unit increase in digital literacy, the probability of drinking more than three times in the past month decreases by 0.3%. The combination of results from columns (4)–(6) reveals that digital literacy can improve physical and mental health by encouraging reductions in alcohol consumption. In summary, digital literacy can improve the physical and mental health of older adults by reducing smoking and drinking behaviors, thus validating Hypothesis 3a.

**Table 11:** Digital literacy, healthy living, and the physical and mental health of older adults.

Variables	(1)	(2)	(3)	(4)	(5)	(6)
	Nonsmoking	ADL	MH	Non-alcoholic	ADL	MH
DL	0.003***	0.024***	−0.106***	0.003***	0.029***	−0.107***
	(0.001)	(0.004)	(0.029)	(0.001)	(0.005)	(0.029)
Nonsmoking		0.169***	−1.282***			
		(0.021)	(0.164)			
Non-alcoholic					0.115***	−1.161***
					(0.034)	(0.182)
Constant		9.429***	40.958***		9.388***	39.029***
		(0.172)	(1.079)		(1.895)	(1.082)
N	22,828	22,371	22,251	22,827	18,028	22,251
*R* ^2^		0.161	0.204		0.681	0.217
Controls	Yes	Yes	Yes	Yes	Yes	Yes
Ind. fixed effect	Yes	Yes	Yes	Yes	Yes	Yes
Time fixed effect	Yes	Yes	Yes	Yes	Yes	Yes

(1)***, **, and *denote 1, 5 and 10% significance levels, respectively. (2) Cluster robust standard errors for clustering at the individual level are in parentheses. (3) Columns (1) and (4) report marginal effects.

Models (4)–(6) were used to test whether digital literacy can improve the physical and mental health of older adults by promoting regular physical activity (Table 12). As shown in column (1), digital literacy significantly increases the probability of physical exercise among the older adults. Specifically, for each one-unit increase in digital literacy, the probability of engaging in physical exercise increases by 3.5%. Combining the results from columns (2) and (3) reveals that digital literacy improves the physical and mental health of older adults by increasing the likelihood of physical activity. Column (4) shows that digital literacy also significantly increases the frequency of weekly exercise among older adults. For every one-unit increase in digital literacy, the frequency of weekly exercise increases by 0.032 on average. Combining columns (5) and (6) further demonstrates that digital literacy can improve both the physical and mental health of the older adults by enhancing the frequency of their weekly physical activity, thereby validating Hypothesis 3b. Combining the findings from Tables 11, 12, it is evident that digital literacy promotes the adoption of healthier lifestyles among older adults, specifically, by reducing the probability of smoking and drinking, and increasing both the probability and frequency of physical activity. These behavioral changes contribute to improvements in physical and mental health, thus confirming Hypothesis 3.

**Table 12:** Digital literacy, physical activity, and physical and mental health in older adults.

Variables	(1)	(2)	(3)	(4)	(5)	(6)
	Exercise	ADL	MH	Exercise frequency	ADL	MH
DL	0.035***	0.047***	−0.104***	0.032***	0.063***	−0.111***
	(0.001)	(0.004)	(0.029)	(0.008)	(0.006)	(0.029)
Exercise		0.090***	−1.074***			
		(0.025)	(0.164)			
Exercise frequency					0.007**	−0.155***
					(0.004)	(0.022)
Constant		10.385***	39.471***	5.876***	8.931***	39.432***
		(0.195)	(1.085)	(0.332)	(2.371)	(1.081)
N	27,799	26,470	22,252	25,987	19,607	22,251
*R* ^2^		0.205	0.217	0.337	0.673	0.217
Controls	Yes	Yes	Yes	Yes	Yes	Yes
Ind. fixed effect	Yes	Yes	Yes	Yes	Yes	Yes
Time fixed effect	Yes	Yes	Yes	Yes	Yes	Yes

(1)***, **, and *denote 1, 5 and 10% significance levels, respectively. (2) Cluster robust standard errors for clustering at the individual level are in parentheses. (3) Columns (1) reports marginal effects.

### 4.4 Heterogeneity analysis

#### 4.4.1 Heterogeneous effects of different educational qualifications

Given that the effects of digital literacy on the physical and mental health of older adults may vary by educational level (39, 89), the sample was divided into two groups: those with primary school education or below, and those with junior high school education or above. The results are presented in Table 13. As shown in Table 13, digital literacy improves the physical and mental health of older adults across different educational levels, with a more pronounced effect on the physical health of those with primary school education or below (Column 1). Similarly, the effect on mental health is more pronounced among older adults with primary school education or below. For every one-unit increase in digital literacy, the depression tendency score decreases by 0.122 for those with primary school education or less, compared to a 0.096 decrease for those with junior high school education or above.

**Table 13:** Heterogeneous effects of educational qualifications.

Variables	(1)	(2)
	ADL	MH
Digital Literacy × Primary and below	0.062***	−0.122***
	(0.006)	(0.042)
Digital Literacy × Junior High School and above	0.038***	−0.096***
	(0.006)	(0.035)
Primary and below	0.121**	0.051
	(0.263)	(0.334)
Constant	10.296***	38.741***
	(0.204)	(1.145)
N	26,470	22,252
*R* ^2^	0.205	0.215
Controls	Yes	Yes
Ind. fixed effect	Yes	Yes
Time fixed effect	Yes	Yes

(1)***, **, and *denote 1, 5 and 10% significance levels, respectively. (2) Cluster robust standard errors for clustering at the individual level are in parentheses.

#### 4.4.2 Heterogeneous gender-specific effects

Given that gender may influence the impact of digital literacy on the physical and mental health of older adults, this study further examines the effects of digital literacy on the physical and mental health of older adults across genders (70, 90). As shown in Table 14, digital literacy effectively improves both the physical and mental health of older adults, but the effects vary by gender. Specifically, digital literacy has a more pronounced impact on the physical health of men (Column 1), for each unit increase in digital literacy, men’s physical health scores rise by 0.053 and women’s by 0.045, while it exerts a more pronounced effect on women’s mental health (Column 2). Each unit increase in digital literacy reduces men’s CES-D depression scores by 0.086 and women’s by 0.134.

**Table 14:** Heterogeneous effects of gender.

Variables	(1)	(2)
	ADL	MH
Digital Literacy × females	0.045***	−0.134***
	(0.007)	(0.039)
Digital literacy × male	0.053***	−0.086***
	(0.006)	(0.033)
Females	−0.019	2.464***
	(0.143)	(0.153)
Constant	8.104***	36.355***
	(1.636)	(1.116)
N	24,174	22,252
*R* ^2^	0.752	0.215
Controls	Yes	Yes
Ind. fixed effect	Yes	Yes
Time fixed effect	Yes	Yes

(1)***, **, and *denote 1, 5 and 10% significance levels, respectively. (2) Cluster robust standard errors for clustering at the individual level are in parentheses. (3) Columns (1) reports marginal effects.

#### 4.4.3 Heterogeneous effects of different physical conditions

Table 15 presents the heterogeneous effects of digital literacy on older adults with different physical conditions (91, 92). The sample was divided into two groups based on whether the older adults had experienced a chronic disease within the past 6 months. The combined results from columns (1) and (2) show that digital literacy significantly improves the physical and mental health of older adults across various health conditions. However, the effect is more pronounced for those with chronic diseases. Specifically, for each one-unit increase in digital literacy, physical health scores for older adults with chronic diseases increase by an average of 0.128, and depression tendency scores decrease by 0.468. In contrast, for those without chronic diseases, the corresponding changes are 0.052 and 0.169, respectively.

**Table 15:** Heterogeneous effects of chronic diseases.

Variables	(1)	(2)
	ADL	MH
Digital literacy × no chronic disease in 6 months	0.052***	−0.169**
	(0.010)	(0.066)
Digital Literacy × chronic illness within 6 months	0.128***	−0.468***
	(0.015)	(0.091)
No chronic diseases within the last 6 months	0.262***	−3.491***
	(0.025)	(0.156)
Constant	8.939***	43.097***
	(2.358)	(0.975)
N	19,607	22,583
*R* ^2^	0.675	0.169
Controls	Yes	Yes
Ind. fixed effect	Yes	Yes
Time fixed effect	Yes	Yes

(1)***, **, and *denote 1, 5 and 10% significance levels, respectively. (2) Cluster robust standard errors for clustering at the individual level are in parentheses. (3) Columns (1) reports marginal effects.

## 5 Discussion

We employed panel data from the CFPS covering the period from 2016 to 2022. By integrating the digital divide framework with the concept of digital literacy, we constructed a multidimensional index to evaluate older adults’ digital literacy, encompassing material access, motivation, and usage skills. This study investigated the influence of digital literacy—both as a composite measure and across its individual dimensions—on the physical and mental health of older adults. Our results demonstrate that digital literacy significantly enhances older adults’ physical and mental well-being, which is consistent with previous studies (12, 16, 17). A more nuanced analysis across dimensions reveals distinct patterns: while both material access and motivation consistently improve physical and mental health, the effects of specific usage skills vary. All four usage types—studying, socializing, entertainment, and shopping—contribute to better physical health; however, only social and entertainment activities show positive effects on mental well-being, with learning- and shopping-related uses demonstrating no significant benefits.

In terms of material access, the use of digital devices broadens older adults’ information and social channels. For example, Cresci et al. (93) found that internet use encourages older adults to browse health-related websites, supporting effective health management. Moreover, internet use significantly enhances social interaction among the older population. As noted by Janssen et al. (94), internet use helps expand personal social networks and foster durable connections, thereby building social capital that supplements older adults’ existing social resources. From a motivational perspective, older adults with more positive attitudes toward digital information technology are better able to improve their physical and mental well-being. This is because individuals with stronger motivation are less likely to experience frustration when first using digital devices and do not perceive devices as an additional source of pressure (24). Instead, they often gain a sense of self-fulfillment, which strengthens their confidence in using technology (95). This confidence, in turn, encourages proactive use of digital health services (82) and promotes scientifically informed preventive health behaviors, ultimately contributing to improved physical and mental health (96). Regarding usage skills, greater proficiency enables older adults to engage in a wider range of online activities—such as knowledge acquisition, entertainment, and economic participation—further enhancing their physical health. Jiang and Luo (31) observed that older adults who use the internet for socializing, information seeking, and learning tend to exhibit better physical health. Similarly, Kyriazis and Kiourti (97) found that online video games can improve cognitive function among older adults, while complex 3D games have also been shown to enhance balance and strengthen lower limb muscles (98). However, not all online activities yield mental health benefits. This study found that online learning and shopping were ineffective in improving older adults’ mental well-being—a result consistent with Nan et al. (99), who suggested that the scarcity of psychologically tailored, self-directed learning resources on digital platforms makes it difficult for older adults to alleviate emotional distress through such means. Similarly, Zheng et al. (100) highlighted potential negative effects of online shopping, noting that exposure to products beyond users’ purchasing capacity can trigger impulse buying and financial stress, thereby inducing negative emotions.

This study identifies two primary mechanisms through which digital literacy improves older adults’ physical and mental health: enhanced social support and healthier lifestyle adoption. First, digital literacy strengthens social support through two complementary pathways. It helps older adults accumulate richer social capital—both online and offline—promoting more active social participation. In terms of offline social capital, stronger kinship networks encourage more frequent social activities, which buffer daily stress, promote physical activity, and enhance self-efficacy, ultimately improving well-being (46, 47). Simultaneously, online social capital built through digital interaction helps develop weak social ties and virtual community participation, strengthening social identity and reducing unhealthy behaviors (46, 101). Additionally, digital literacy strengthens intergenerational connections, which encourage physical activity—improving physical health (52, 53)—while also supporting cognitive function and subjective well-being (52).

Second, digital literacy promotes healthier behaviors, particularly smoking and alcohol cessation. Digital health tools—such as tailored text messages, apps, and websites—provide personalized support for reducing tobacco and alcohol use. These platforms help users set goals, deliver relevant health information, and suggest alternative behaviors to manage cravings. For instance, Fahey et al. (102) found that digital smoking cessation interventions nearly doubled success rates compared to traditional methods, with outcomes improving as app engagement increased. Similarly, Masaki et al. (103) reported a 7% rise in cessation success for each program module completed within a week. Digital alcohol interventions also show significant effects: Kaner et al. (104) observed a reduction of three standard drinks per week among participants, and Bertholet et al. (105) found that using a drinking-control app reduced both drinking days and heavy drinking episodes. Furthermore, digital literacy encourages more frequent and consistent physical exercise. Regular activity improves cardiovascular health, blood pressure, and lipid profiles (106, 107), while resistance training preserves muscle mass and functional independence (108, 109). Exercise also enhances sleep quality, self-efficacy, and mental well-being (92, 110, 111). By facilitating access to exercise guidance and motivation, digital literacy helps integrate regular physical activity into older adults’ lives, further supporting their overall health.

The study further reveals that the health benefits of digital literacy vary across subgroups defined by educational attainment, gender, and baseline health status. First, the positive effects are more pronounced among older adults with lower educational attainment. This group tends to rely more heavily on family and friends for information, so improved digital literacy significantly expands their social circles and engagement, thereby enhancing mental well-being (8). In contrast, highly educated older adults typically already possess broader social networks, making the marginal benefit of digital literacy on social participation and psychological health relatively smaller (11). Second, the benefits of digital literacy differ among older adults by gender. It more effectively improves physical health among men, likely because they often engage in goal-oriented health management and fitness activities, where digital devices support healthier behaviors (90). Among women, digital literacy shows stronger effects on mental health, as they are more inclined to use diverse online social platforms to expand social networks, alleviating loneliness and depressive symptoms (69, 112, 113). Finally, the health benefits are particularly strong among older adults with pre-existing health conditions. This group tends to be more proactive in seeking health information, managing care, and adopting healthier lifestyles (55). Additionally, online health communities provide peer support, enhance social belonging, and offer valuable resources, further improving both physical and mental well-being in this vulnerable subgroup.

## 6 Limitations

Although our findings provide new actionable recommendations for advancing healthy aging, certain limitations remain. Constrained by data availability, we are unable to fully explore the impact of older adults’ usage characteristics on digital information platforms (such as content creation, usage applications and diversity, cybersecurity awareness, and other characteristics) upon their physical and mental health. Future research could design specialized questionnaires targeting older adults’ digital usage patterns and incorporate objective assessment methods (e.g., recruiting older adult volunteers with varying educational levels to participate in digital skills tests) to measure older adults’ digital literacy from both subjective and objective dimensions.

## 7 Conclusion

Since the 21st century, China has rapidly transitioned into an aging society, leading to a growing demand for healthcare services for the older population. Ensuring that older adults maintain good physical and mental health while preserving their independence and quality of life has become a critical issue in addressing the challenges of population aging. In this context, this paper provides a comprehensive assessment of the digital literacy of older adults across three dimensions: material access, motivation, and usage skills, using panel data from the CFPS (2016–2022). The study explores the impact of digital literacy on the physical and mental health of older adults and investigates the mechanisms through which digital health literacy enhances these outcomes, focusing on social support and healthier lifestyles. The study also examines the heterogeneous effects of digital literacy on the physical and mental health of older adults from the perspectives of education, gender, and physical condition. The findings reveal that digital literacy significantly improves both the physical and mental health of older adults. Improvements in digital literacy across the three dimensions, namely, material access, motivation, and usage skills, effectively enhance physical and mental health. These conclusions remain robust after a series of robustness tests. Further, the study shows that digital literacy improves the physical and mental health of older adults by enhancing social support and promoting healthier lifestyles. From the social support perspective, digital literacy not only broadens older adults’ online and offline social networks and strengthens their social capital, but it also significantly increases the frequency of interactions between older adults and their children, fostering closer intergenerational relationships and improving both physical and mental health. From the healthy lifestyle perspective, digital literacy effectively reduces the probability of smoking and drinking among older adults while increasing the likelihood and frequency of regular physical activity, thereby improving their overall well-being. Additionally, the effect of digital literacy on the physical and mental health of older adults is heterogeneous, varying according to education, gender, and health status. The health benefits of digital literacy are more substantial for older adults who have lower educational levels or chronic diseases. Moreover, these benefits demonstrate a gender-specific pattern: the improvement in physical health is more significant among men, whereas the enhancement in mental health is more marked among women. Our findings have several important policy implications.

First, a multi-party collaborative mechanism for enhancing the digital literacy of older adults should be established. Government departments must integrate the enhancement of digital literacy for older adults into national strategies. This should include promoting the comprehensive application of next-generation information technologies, such as the Internet, big data, and the Internet of Things, in healthcare for older adults. The goal is to innovate service delivery models for intelligent services and significantly improve the effectiveness of health management services. Building on the national health information platform, a collaborative management platform for aging health information should be developed. This platform should facilitate the integration of multi-source data and ensure cross-system interconnectivity, providing full-process digital support for accurate health services tailored to older adults.

Second, efforts should be made to improve the supply of aging-friendly services through digital products and enhance the overall user experience with digital technologies. The provision of intelligent devices with aging-friendly functions should be expanded, the barrier-free transformation of aging-friendly applications should be promoted, and a coordinated effort should be made to advance both online and offline services. This approach will ensure more accessible and balanced digital products and services for the older population.

Third, a robust, multidimensional capacity-building mechanism for social support and health behaviors is essential. A systematic communication matrix using new media platforms, such as short videos and WeChat public accounts, should be established to promote geriatric health knowledge and highlight exemplary cases. The development of region-specific practice paradigms should be encouraged to improve the effectiveness of older adults’ participation in health education. A positive interaction mechanism of “silver hair driving silver hair” should be constructed. The standardization of age-friendly fitness facilities in both urban and rural communities and healthcare institutions should also be prioritized, with a focus on increasing the coverage of barrier-free exercise spaces. Moreover, the development of evidence-based age-friendly sports and leisure programs, the formulation of a Scientific Fitness Guidance Manual for Seniors, and the establishment of a system for grading and adapting exercise intensity are critical. These efforts should be integrated into chronic disease prevention and treatment guidelines, fostering a seamless connection between rehabilitation and active health management.

Lastly, measures to improve the physical and mental health of the older adults should be tailored and differentiated. Community-based channels for social participation in digital information technology learning should be provided. “Mobile phone classrooms” in neighborhoods can be set up, where community staff or young volunteers teach digital skills to older adults. Additionally, digital skills training should be made more accessible in local communities, making everyday life easier for the older population. Online geriatric health education programs should also be encouraged, including courses on healthcare and caregiving skills for older adults, their families, and caregivers.

## Data Availability

Publicly available datasets were analyzed in this study. This data can be found here: isss.pku.edu.cn/cfps/index.htm.

## Footnotes

1 Source: WHO, Aging and Health, see: https://www.who.int/news-room/fact-sheets/detail/ageing-and-health

2 Source: China Statistical Yearbook (2024), see: https://www.stats.gov.cn/sj/pcsj/rkpc/6rp/html/B0802c.htm

3 Source: China Ageing Development Report (2024), see: https://www.pishu.com.cn/skwx_ps/bookdetail?SiteID=14&ID=15740411

4 Source: The 55th Statistical Report on Internet Development in China.
